# EphA4 Regulates the Balance between Self-Renewal and Differentiation of Radial Glial Cells and Intermediate Neuronal Precursors in Cooperation with FGF Signaling

**DOI:** 10.1371/journal.pone.0126942

**Published:** 2015-05-15

**Authors:** Qingfa Chen, Daiki Arai, Kazuki Kawakami, Takahiro Sawada, Xuefeng Jing, Masayasu Miyajima, Syu-ichi Hirai, Kazushige Sakaguchi, Kenryo Furushima

**Affiliations:** 1 Department of Molecular Cell Biology and Molecular Medicine, Institute of Advanced Medicine, Wakayama Medical University, 811–1 Kimiidera, Wakayama, 641–8509, Japan; 2 Laboratory Animal Center, Wakayama Medical University, 811–1 Kimiidera, Wakayama, 641–8509, Japan; 3 Department of Biology, Wakayama Medical University, 580 Mikazura, Wakayama, 641–0011, Japan; University of Wurzburg, GERMANY

## Abstract

In mouse cerebral corticogenesis, neurons are generated from radial glial cells (RGCs) or from their immediate progeny, intermediate neuronal precursors (INPs). The balance between self-renewal of these neuronal precursors and specification of cell fate is critical for proper cortical development, but the signaling mechanisms that regulate this progression are poorly understood. EphA4, a member of the receptor tyrosine kinase superfamily, is expressed in RGCs during embryogenesis. To illuminate the function of EphA4 in RGC cell fate determination during early corticogenesis, we deleted *Epha4* in cortical cells at E11.5 or E13.5. Loss of EphA4 at both stages led to precocious in vivo RGC differentiation toward neurogenesis. Cortical cells isolated at E14.5 and E15.5 from both deletion mutants showed reduced capacity for neurosphere formation with greater differentiation toward neurons. They also exhibited lower phosphorylation of ERK and FRS2α in the presence of FGF. The size of the cerebral cortex at P0 was smaller than that of controls when *Epha4* was deleted at E11.5 but not when it was deleted at E13.5, although the cortical layers were formed normally in both mutants. The number of PAX6-positive RGCs decreased at later developmental stages only in the E11.5 *Epha4* deletion mutant. These results suggest that EphA4, in cooperation with an FGF signal, contributes to the maintenance of RGC self-renewal and repression of RGC differentiation through the neuronal lineage. This function of EphA4 is especially critical and uncompensated in early stages of corticogenesis, and thus deletion at E11.5 reduces the size of the neonatal cortex.

## Introduction

During corticogenesis, radial glial cells (RGCs) reproduce in the apical ventricular zone (VZ) and differentiate into intermediate neuronal precursors (INPs) during early stages, and then into several types of neuronal cells at later stages of embryonic development [1, 2]. INPs generated from RGCs divide once or twice in the basal VZ or in the subventricular zone (SVZ) to generate more INPs (self-renewal) or post-mitotic neurons [3]. Neuronal cells generated from RGCs or INPs migrate to the cortical plate in an inside-out laminar pattern to form the six cortical layers [4, 5]. The neurons in deeper cortical layers (5/6) are generated directly from RGCs or indirectly via INPs, whereas the neurons in the upper cortical layers (2/3 to 4) are generated exclusively from INPs [6]. As a result, mammalian cortex generates six layers by segregating specific neuronal cells. RGCs, INPs, and neuronal cells in each layer can be identified and traced during corticogenesis by the sequential expression of specific transcription factors [7–9]. Intriguingly, early loss of INPs leads to a decrease in cortical surface expansion and thickness, with a reduction in neuronal number in all cortical layers [6], suggesting that INP progeny contribute to the correct morphogenesis of each cortical layer.

Fibroblast growth factors (FGFs) promote RGC proliferation via phosphorylation of FRS2α and ERK [10–13], but it is unclear how they exert their effects on RGCs and neuronal progenitor cells *in vivo* and how the FGF signal induces the RGC-to-neuronal cell transition. Simultaneous deletion of three FGF receptor genes (*FGFR1*, *FGFR2*, and *FGFR3*) during cortical neurogenesis depletes RGCs due to precocious neurogenesis [14, 15], possibly mediated by changes in Notch signaling downstream of the FGF signal [15].

EphA4 is a member of the Eph receptor tyrosine kinase family and is activated by all members of the ephrin-A ligand family and most members of the ephrin-B family [16, 17]. We previously demonstrated that direct binding of EphA4 to FGF receptors (FGFRs) leads to activation of FRS2α and MAPK [18], suggesting possible regulation of FGF signal transduction in RGCs by EphA4. EphA4 is expressed in the neural ectoderm at embryonic day 8 (E8.0) and then in various cell types throughout the cortex during subsequent stages of neurodevelopment [19, 20]. EphA4 is a well-known neuronal repellant that regulates the formation of the axon tract and cortical network as well as radial migration of cortical neurons in the mouse cortex [21–24]. EphA4 maintains neural stem cells in the adult cortex and *Epha4* null mice exhibit a thinner cortex than wild-type mice and reduced proliferation of cortical RGCs [25, 26]. However, little is known of the cell- and stage-specific function of EphA4 in corticogenesis. In particular, it is unclear whether EphA4 contributes to proliferation and/or differentiation of neural stem/progenitor cells.

Here we studied the stage-specific functions of EphA4 in corticogenesis by creating two conditional *Epha4* knockout mice in which the gene was deleted at different developmental stages.

## Materials and Methods

### Mice

The *Epha4* ^*flox/flox*^ [27], *Nestin-Cre* [28], and *hGFAP-Cre* [29] mice have been described previously and were genotyped accordingly. The morning the vaginal plug was detected was defined as embryonic day 0.5 (E0.5). Pups born on the 19th day after plug detection were defined as postnatal day 0 (P0) mice. This study was carried out in strict accordance with the recommendations in the Guide for the Care and Use of Laboratory Animals of the National Institutes of Health. All experiments were performed in accordance with the regulations of the Wakayama Medical University Animal Care and Use Committee. The protocols were approved by the committee (permit numbers: 23–30, 23–34, and 23–49). All surgery was performed under sodium pentobarbital anesthesia and all efforts were made to minimize animal suffering.

### Immunohistochemistry and Nissl staining

Whole mouse heads or isolated brains retrieved between E10.5 and P0 were fixed overnight in 4% paraformaldehyde (PFA) at 4°C and then embedded in paraffin wax. Paraffin sections (6-μm-thick) were de-waxed, hydrated, heated at 121°C for 1 min in 10 mM sodium citrate (pH 6.0) for antigen retrieval, and then immunohistochemically stained using a standard protocol. Briefly, sections were pretreated with 10% goat serum in Tris-buffered saline and Tween 20 (pH 8.0) at room temperature (RT) for 30 min, and incubated with a primary antibody at 4°C overnight. The primary antibodies used in this study were as follows: rabbit anti-EphA4 (Cat. #sc-921, 1:300, Santa Cruz), mouse anti-pHH3 (anti-phosphorylated histone H3, Cat. #ab14955, 1:600, Abcam), rabbit anti-Class III β-tubulin (TUBB3) (Cat. #MRB-435P, 1:1000, Covance), mouse anti-TUBB3 (Cat. #MMS-435P, 1:1000, Covance), rabbit anti-TBR2 (Cat. #AB2283, 1:1000, Millipore; Cat.#ab23345, 1:500, Abcam), rabbit anti-PAX6 (Cat. #PRB-278P, 1:600, Covance), rabbit anti-Ki67 (Cat. #ab16667, 1:200, Abcam), rabbit anti-CUX1 (Cat. #sc-13024, 1:100, Santa Cruz), rat anti-CTIP2 (Cat. #ab18465, 1:1000, Abcam), and mouse anti-BrdU (Cat. #MI-11-3, 1:1000, MBL). After a wash in PBS, immunolabeled sections were incubated at RT for 1 h with secondary antibodies (Vector Labs) conjugated to horseradish peroxidase for 3,3’-diaminobenzidine staining or to Alexa Fluor 488 and Alexa Fluor 568 (Molecular Probes) for immunofluorescence staining. Sections were then washed in PBS, counterstained with 4’,6’-diamino-2-phenylindole (DAPI; Molecular Probes), and mounted with Gel/Mount (DAKO). Staining was detected with a Keyence BZ-9000 microscope or a Zeiss LSM5 Pascal confocal microscope.

For Nissl staining, whole mouse heads or isolated brains were fixed as described above, and paraffin sections (10-μm-thick) were stained with 0.1% cresyl violet [30]. Staining was detected with a Keyence BZ-9000 microscope.

### Histochemical and immunohistochemical quantification

**Fig 1:**
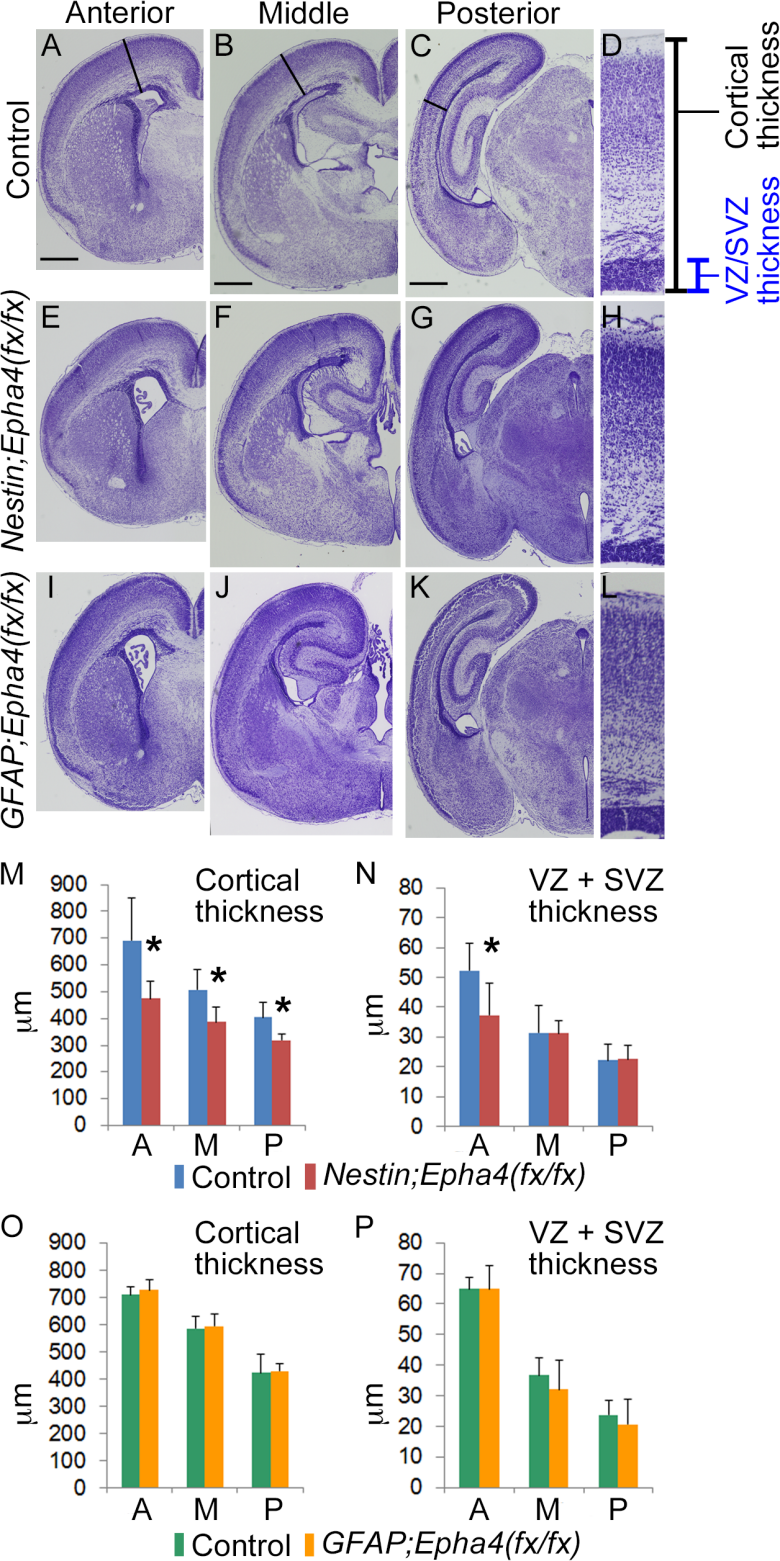
Effect of stage-dependent EphA4 deletion on the cerebral cortex at P0. **A–L**, Cresyl violet stain of coronal brain sections from control (A–D), *Nestin*;*Epha4* ^*fx/fx*^ (E–H), and *GFAP*;*Epha4* ^*fx/fx*^ (I–L) mice. Shown are the corresponding sections of these mice along the antero-posterior axis of the telencephalon. The *Nestin*;*Epha4* ^*fx/fx*^ cortex appears smaller than the control cortex, while the *GFAP*;*Epha4* ^*fx/fx*^ cortex is almost the same size as the control cortex. **M–P,** Quantification of cortical thickness (M, O) and proliferating zone (VZ+SVZ) width (N, P) in *Nestin;Epha4* ^*fx/fx*^ (M, N) and *GFAP;Epha4* ^*fx/fx*^ (O, P) mice. Solid lines in control animals (A to C) and corresponding lines in mutants (E–G, I–K) indicate the antero-posterior location where these thicknesses were measured. Enlargements of the measured areas in the anterior part (A, E, and I) are shown in the rightmost column (D, H, and L, respectively). Cortical thickness was reduced in all brain sections from *Nestin*;*Epha4* ^*fx/fx*^ mice, and the proliferating zone width was thinner only in anterior sections. However, in *GFAP*;*Epha4* ^*fx/fx*^ mice, cortical thickness and proliferating zone width were similar to controls. A, anterior; M, middle; P, posterior. N = 5 for each measurement, (*) P < 0.05. Error bars represent SD. Scale bar, 500 μm.

Coronal brain sections were prepared as described to measure cortical thickness, layer morphology, and the location and number of specific cell populations (RGCs, INPs, and their progeny) at three locations along the anterior-posterior axis: anterior, the section where the corpus callosum crosses the hemispheres and the hippocampal commissure appears; middle, the section that has both the medial-lateral habenular and ventral-lateral thalamic nuclei; posterior, the section just posterior to the ventral-lateral thalamic nucleus. Values at each location are the averages of three sections from 3–5 control and mutant littermate pairs (9–15 sections). The thickness of the P0 cortex was measured midway between the pallial–subpallial ventricular zone boundary and the dorsalmost bend of the cortical VZ at the interhemispheric fissure (demarcated by the bars in Fig 1). The thickness of the proliferative zone was also determined at the same position. The thicknesses of the proliferating layer (PAX6 immunoreactive) and the neuronal layer (TUBB3 immunoreactive) were determined at the same anterior coronal position. The numbers of PAX6-expressing (+) cells, TBR2+ cells, pHH3+ cells, BrdU-labeled (BrdU+) cells, BrdU+Ki67+ cells, BrdU+TUBB3+ cells, BrdU+CUX1+ cells, and BrdU+CTIP2+ cells were counted within 100–300-μm-wide rectangular regions. At P0 and E17.5, the rectangular regions correspond to the mid-area of the anterior somatosensory cortex and, from E13.5 to E15.5, the midway of the regions correspond to the midway between the pallial–subpallial VZ boundary and the dorsal VZ bend. The stained cells were generally counted in a 100-μm-wide radial segment of the entire cortical thickness, but the number of pHH3+ cells with PAX6+ or TBR2+ were counted in a 300-μm-wide radial segment of the entire cortical thickness and normalized to 100 μm.

### BrdU and TUNEL labeling

Pregnant dams received an intraperitoneal injection of BrdU, 50 mg/kg body weight. For cell cycle exit assays, pregnant females carrying E13.5 or E14.5 embryos were injected with BrdU and the embryos were collected at E14.5 and E15.5, respectively, and processed for immunohistochemistry. For birthdating assays, pregnant females carrying E11.5, E13.5, E15.5, or E17.5 embryos were injected with BrdU and embryos were collected at P0. Sections were denatured in 1.5 N HCl for 30 min at 37°C, followed by neutralization in 0.1 M sodium borate (pH 8.5) before incubation with the primary antibodies. The cortical thickness was divided into 10 parallel subregions (bins) and the number of BrdU+ cells was counted in each bin from three sections per mouse. At least three mice were examined for each genotype. TUNEL staining was performed following the manufacturer’s protocol (Takara). Cells with a condensed shape and dark black staining were regarded as apoptotic and were easily distinguished from blood cells, which showed no shrinkage and/or were stained brown only in the cytoplasm following the TUNEL protocol.

### Neurosphere cultures

Neurospheres were derived from single cell suspensions obtained by trypsin digestion of E14.5 cortices. Cells were plated at clonal density (< 1 × 10^5^ cells/ml) in basal serum-free media (DMEM/F12 supplemented with B27, 25 mM HEPES, 0.6% glucose, and 1% pen/strep) containing FGF2 (20 ng/ml) and EGF (20 ng/ml), maintained at 37°C, and supplemented with fresh media every other day. For immunohistochemical analysis of neurosphere differentiation using anti-TBR2 and-TUBB3 antibodies, primary neurospheres grown for 4–5 days were trypsinized and single cells were plated and cultured in the same medium for 24 h at a density of 1 × 10^5^ cells/well in 8-well chamber slides coated with 0.01% PLL and 200 μg/ml fibronectin. The cells were fixed with 4% PFA for 30 min at RT and treated with 0.01% Triton X-100/PBS. The cells were blocked with 1% goat serum in PBS at RT for 30 min and incubated with TBR2 and TUBB3 antibodies at 4°C overnight. The cells were incubated at RT for 1 h with Alexa Flour 568 secondary antibody and counterstained with DAPI and mounted with Gel/Mount. For neurosphere formation, following the third passage, single cells (dissociated by trypsin treatment) were replated and cultured in 96-well plates at clonal density in basal serum-free media (DMEM/F12 supplemented with B27, 0.6% glucose, and 1% pen/strep) containing vehicle, FGF2 (20 ng/ml) alone, clustered ephrinA1-Fc (0.5 μg/ml) alone, or FGF2 plus clustered ephrinA1-Fc. Ephrin-A1 fused to human IgG-Fc was purchased from Sigma-Aldrich. Before application, 5 μg of ephrin-A1-Fc was incubated with 12 μg of rabbit anti-human IgG-Fc (Jackson ImmunoResearch) in 1 ml of PBS at 4°C for at least 1 h to produce clustered ephrin-A1 [18]. The number of tertiary neurospheres was determined under light microscopy.

### Western blotting

Telencephalic cortices were dissected from E10.5 to P0 mouse brains. For FGF2 stimulation experiments at E15.5, cells from isolated telencephalic cortices were mechanically dissociated and incubated in DMEM supplemented with 0.6% glucose, 25 mM HEPES, and 1% penicillin-streptomycin (pen/strep) for 60 min at 37°C under a 5% CO_2_ atmosphere. The cell suspension was then divided into two tubes, one control and the other incubated with FGF2 (100 ng/ml) for 20 min. Treated and control cells were lysed in buffer containing 50 mM HEPES, 1% Triton X-100, 5 mM ethylenediaminetetraacetic acid, 50 mM NaCl, 10 mM sodium pyrophosphate, 50 mM sodium fluoride, 1 mM sodium orthovanadate, and protease inhibitors (1 mM phenylmethylsulfonyl fluoride, 1 μM aprotinin, 1 μM leupeptin, and 1 μM pepstatin A). Proteins were fractionated by sodium dodecyl sulfate-polyacrylamide gel (SDS-PAGE) electrophoresis (15 μg–50 μg protein per gel lane) and blotted onto polyvinylidene fluoride membranes (Millipore, Billerica, MA, USA). Immunodetection was performed with an Immobilon Western Blotting Detection System (Millipore). Antibodies used for western blotting were as follows: rabbit anti-EphA4 polyclonal antibody (Cat. #sc-921; 1:2000, Santa Cruz), rabbit anti-FRS2α polyclonal antibody (Cat. #sc-8318; 1:1000, Santa Cruz), rabbit anti-GAPDH (Cat. #sc-25778, 1:3000, Santa Cruz), rabbit anti-phospho-p44/42 polyclonal mitogen-activated protein kinase (MAPK) (ERK) (Thr202/Tyr204) antibody (Cat. #9101; 1:4000, Cell Signaling Tech.), rabbit anti-p44/42 MAPK (ERK) polyclonal antibody (Cat. #9102; 1:4000, Cell Signaling Tech.), and rabbit anti-pFRS2α(Y196) (Cat. #3861; 1:1000, Cell Signaling Tech.). Band intensities were quantified using CS Analyzer version 3.0 (ATTO Corporation). Because the signal intensity of FRS2α was very weak, pFRS2α was normalized to the signal intensity of GAPDH, whereas the intensity of pERK was normalized to that of ERK. Primary neurospheres were isolated as described above and used for western blot analysis of TBR2 and TUBB3.

### Statistical analysis

All values are expressed as mean ± standard deviation (SD). Error bars in graphs represent the SD. Numerical group means were compared by Student’s t-tests. For all experiments comparing mutants to controls, values from 3–5 pairs of littermates were averaged for each measurement location.

## Results

### Stage-dependent effect of radial glia *EphA4* deletion on cortical size

**Fig 2:**
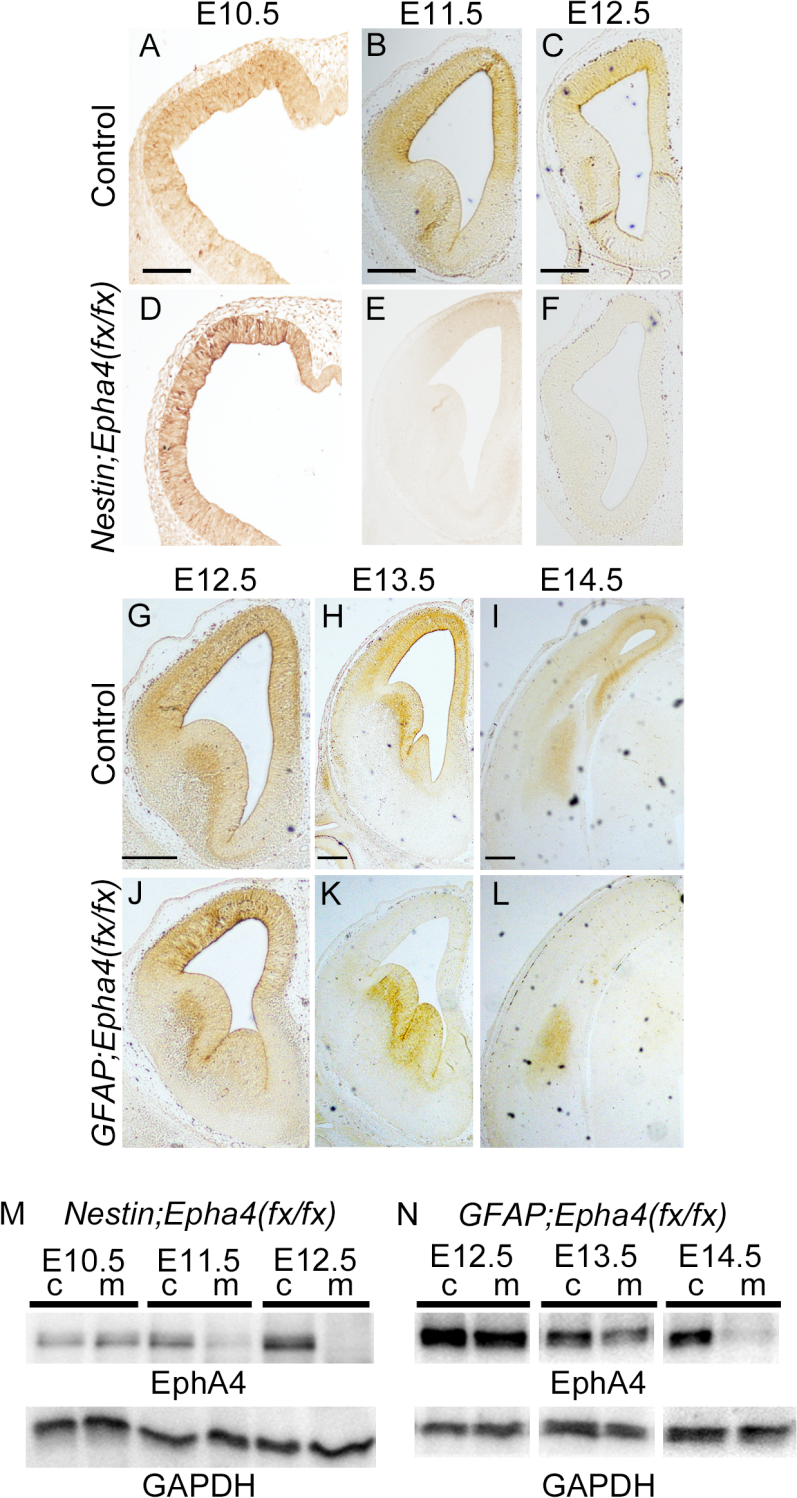
*Nestin-Cre*- and *hGFAP-Cre*-mediated deletion of *Epha4* in the developing forebrain at E11.5 and E13.5, respectively. **A–F**, Immunohistochemical EphA4 expression pattern in control (A–C) and *Nestin*;*Epha4* ^*fx/fx*^ mouse brain (D–F). Expression of EphA4 in *Nestin*;*Epha4* ^*fx/fx*^ mouse forebrain starts to disappear after E11.5. **G–L,** Immunohistochemical EphA4 expression pattern in control (G–I) and *GFAP*;*Epha4* ^*fx/fx*^ mouse brain (J–L). Expression of EphA4 in *GFAP*;*Epha4* ^*fx/fx*^ mouse cortex starts to diminish after E13.5 but does not disappear completely in this mutant. **M, N,** western blot analysis of EphA4 expression in the cortex of control and *Nestin;Epha4* ^*fx/fx*^ mice (M) or *GFAP;Epha4* ^*fx/fx*^ mice (N). Western blotting results are consistent with those of immunohistochemistry. c, control; m, *Nestin;Epha4* ^*fx/fx*^ in panel M and *GFAP;Epha4* ^*fx/fx*^ in panel N. Scale bars, 100 μm.

To investigate the role of EphA4 in the temporal control of corticogenesis, we conditionally deleted *Epha4* in RGCs of the cortex before the onset of deep cortical layer formation or upper layer formation. Targeted deletion of *Epha4* was accomplished via Cre-dependent recombination of the loxP-flanked sequences in *Epha4* ^*fx*^ alleles. Mice carrying *Epha4* ^*fx*^ alleles were crossed with mice carrying *Nestin-Cre* or *hGFAP-Cre* transgenes to generate mice homozygous for *Epha4* ^*fx*^ alleles and *Cre* under the control of the nestin or glial fibrillary acid protein (GFAP) promoter (*Nestin*;*Epha4* ^*fx/fx*^ and *GFAP*;*Epha4* ^*fx/fx*^). Mice carrying homozygous *Epha4* ^*fx*^ alleles were used as controls. The *Nestin* or *hGFAP* promoter directs expression of Cre recombinase specifically in RGCs of the dorsal telencephalon and cerebral cortex starting at E11.5 or E13.5, respectively, resulting in recombined floxed alleles of *Epha4* in most or all RGCs and their progeny [28, 29, 31]. Immunohistochemistry of the forebrain and western blot analysis of the cortex from E10.5 to P0 revealed that most EphA4 expression disappeared at E11.5 in *Nestin*;*Epha4* ^*fx/fx*^ mice and at E13.5 in *GFAP*;*Epha4* ^*fx/fx*^ mice, consistent with the Cre expression pattern (Fig 2). In *GFAP;Epha4* ^*fx/fx*^ mice, EphA4 was expressed abundantly in the basal ganglia, as revealed by immunostaining, and modest expression was also detected in the cortex after E13.5. It has been demonstrated that this extent of reduction in cortical EphA4 expression is sufficient to affect neurogenesis [14, 32, 33]. This approach, using two types of *Epha4* mutants, allowed us to analyze the function of EphA4 during two developmental stages of corticogenesis.

Cresyl violet staining of brain sections revealed that the gross layered structure of the cerebral cortex in *Nestin*;*Epha4* ^*fx/fx*^ mice was normal at P0. However, the size of each hemisphere was reduced slightly compared to control littermates (Fig 1A–1H) and there was a significant reduction in the radial thickness of the entire cortex (Fig 1M). Furthermore, the thicknesses of the VZ and SVZ, considered cortical proliferative zones, were also significantly reduced in the anterior cortical region (Fig 1N). The gross structure of the cortex in *GFAP*;*Epha4* ^*fx/fx*^ mice (engineered for later *Epha4* deletion) was also normal but, in contrast to *Nestin*;*Epha4* ^*fx/fx*^ mice, there were no significant differences in the radial thickness of the cortex or proliferative zones compared to control littermates (Fig 1A–1D, 1I–1L, 1O and 1P). This specific reduction in the entire cortical and anterior proliferative zone thickness may reflect the anterior–posterior developmental gradient of corticogenesis [34]. We further studied the biological effects of EphA4 deletion on corticogenesis by comparing the locations, numbers, and lineages of specific cell populations between *Nestin*;*Epha4* ^*fx/fx*^ and *GFAP*;*Epha4* ^*fx/fx*^ mice. The heterozygotes, *Nestin*;*Epha4* ^*+/fx*^ and *GFAP*;*Epha4* ^*+/fx*^, did not show any difference from controls in the radial thickness of the cortex and proliferative zones (data not shown).

### Loss of EphA4 leads to transient overproduction of cortical INPs and neurons

One possible explanation for the reduced cortex thickness and smaller anterior cortical proliferative zone at P0 in *Nestin;Epha4* ^*fx/fx*^ mouse brain is a reduction in the number of precursor cells such as RGCs and INPs in the proliferative zone, which, aside from directly reducing proliferative zone thickness, would also reduce the pool of precursors needed for populating the cortex with mature neurons. To examine if indeed the *Epha4* deletion reduces the number of RGCs and INPs, we first examined changes in the expression of the transcription factor PAX6, a marker for RGCs [8], from E13.5 to P0. We also examined the effect of *Epha4* deletion on RGC-derived neurogenesis by measuring the expression of the neuronal marker βIII tubulin (TUBB3) during this same embryonic period.

**Fig 3:**
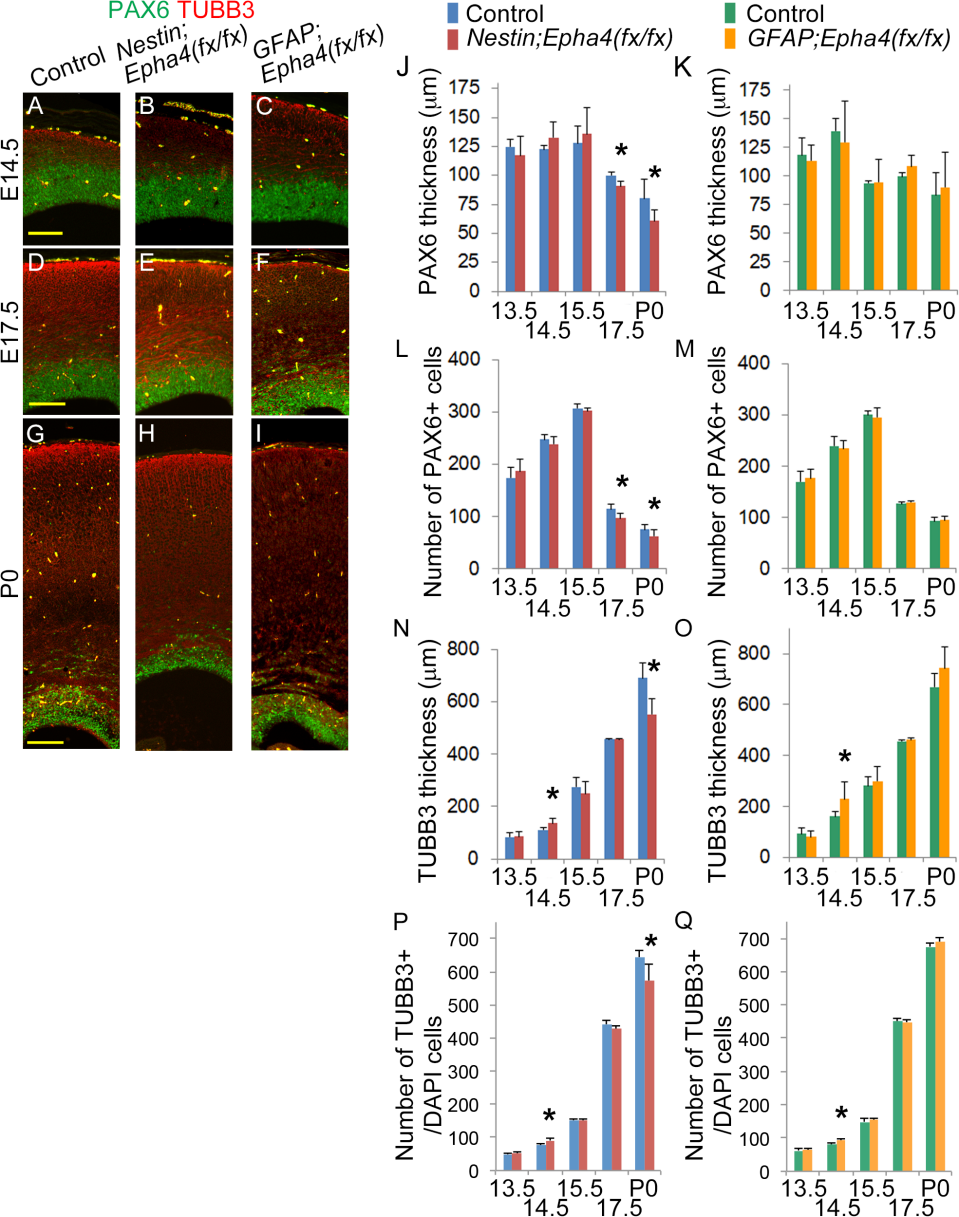
RGC proliferation and differentiation in the cerebral cortex. **A–I**, Anterior coronal sections from mutant and control mice were immunostained for a marker of RGCs (PAX6, green) and for a marker of young differentiated neurons (βIII tubulin [TUBB3], red). Representative sections at E14.5, E17.5, and P0 are shown on the left. **J–Q**, Quantified thickness of the RGC layer (PAX6+, green) (J, K), the number of RGCs (L, M), the differentiated neuronal area (TUBB3+, red) (N, O), and the number of neuronal cells (P, Q) using these stained sections (*Nestin;Epha4* ^*fx/fx*^, J, L, N, P; *GFAP;Epha4* ^*fx/fx*^, K, M, O, Q). The PAX6-expressing progenitor cell population in the VZ was of comparable size in both mutants and controls from E13.5 to E15.5 (A–C, J–M), but was considerably smaller at E17.5 and P0 in *Nestin;Epha4* ^*fx/fx*^ mice (D, E, G, H, J, L). Both mutants showed normal cortex at E13.5, but the cortical layer size of post-mitotic neurons (TUBB3+) became thicker at E14.5 compared to controls (A–C, N–Q). The neuronal area in mutants returned to the size of controls at E15.5 (D–F, N–Q). However, in *Nestin;Epha4* ^*fx/fx*^ mice, the neuronal area was considerably smaller at P0, while in *GFAP;Epha4* ^*fx/fx*^ mice it was not (G–I, N–Q). N = 5, (*) P < 0.05. Error bars represent SD. Scale bar, 100 μm.

The thickness of the PAX6+ cortical area and the number of PAX6+ cells were both normal in *Nestin;Epha4* ^*fx/fx*^ mice from E13.5 to E15.5, but reduced at E17.5 and thereafter compared to controls (Fig 3A, 3B, 3D, 3E, 3G, 3H, 3J and 3L). The thickness of the cortical layer and number of post-mitotic neurons (TUBB3+) were increased at E14.5 in these mutants compared to control littermates (Fig 3A, 3B, 3N and 3P), had about the same thickness and number of controls at E15.5 and E17.5, but were considerably reduced at P0 (Fig 3D, 3E, 3G, 3H, 3N and 3P). This reduced thickness of the TUBB3+ layer and the lower number of TUBB3+ cells at P0 is consistent with the thinner cortex shown in Fig 1. In contrast, the cortical thickness and number of post-mitotic neurons of *GFAP;Epha4* ^*fx/fx*^ mice were normal at P0. We investigated why an approximately 2-day delay in *Epha4* deletion between *Nestin;Epha4* ^*fx/fx*^ and *GFAP;Epha4* ^*fx/fx*^ mice ameliorated this reduction in the cortical thickness and number of neurons in the anterior region by examining the time course of RGC proliferation and differentiation in *GFAP;Epha4* ^*fx/fx*^ mice. The cortical layer of neurons was thicker and the number of neurons was increased at E14.5 in *GFAP;Epha4* ^*fx/fx*^ mice, as shown by TUBB3 staining, then returned to the size of the control after E15.5 without reduction at P0 (Fig 3A, 3C, 3D, 3F, 3G, 3I, 3O and 3Q). In addition, unlike *Nestin;Epha4* ^*fx/fx*^ mice, the thickness of the PAX6+ area and the number of PAX6+ cells in *GFAP;Epha4* ^*fx/fx*^ mouse anterior cortex were not significantly different from controls after E15.5 (Fig 3A, 3C, 3D, 3F, 3G, 3I, 3K and 3M). In contrast to *Epha4* ^*fx/fx*^ mutants, *Nestin*;*Epha4* ^*+/fx*^ and *GFAP*;*Epha4* ^*+/fx*^ mice showed similar radial thickness of TUBB3+ and PAX6+ layers to controls (data not shown). Because there was no difference between heterozygotes and controls, we further compared the phenotypes of homozygotes and controls.

**Fig 4:**
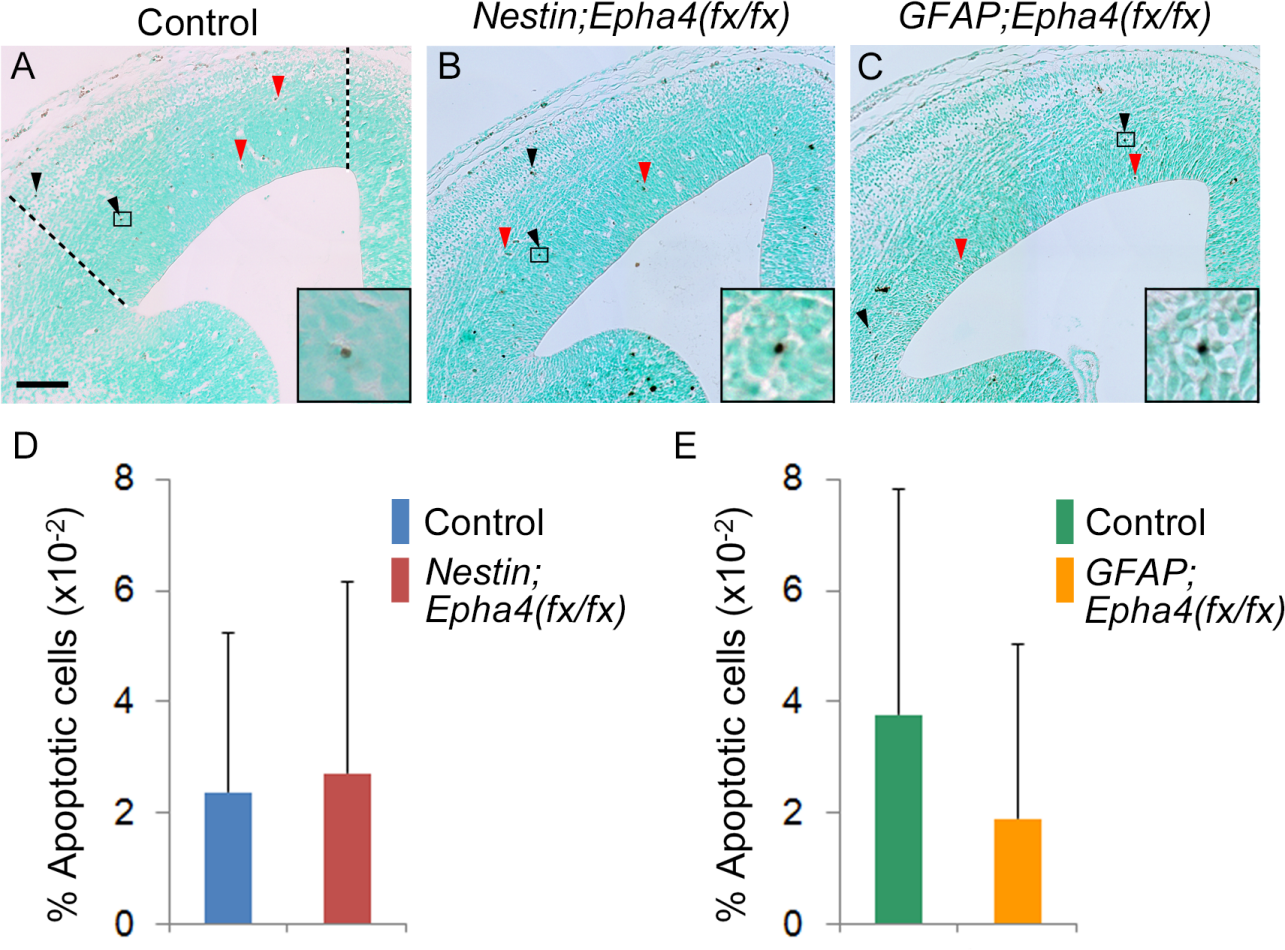
Apoptosis in the cortex. **A–C,** Representative image of apoptotic cells in the cortex of control (A), *Nestin;Epha4* ^*fx/fx*^ (B), and *GFAP;Epha4* ^*fx/fx*^ (C) mice. Apoptotic cells were detected by TUNEL staining at E14.5. Apoptotic cells (black arrowheads) show intense dark black staining, whereas blood cells (red arrow head) exhibit light brown staining in the cytoplasm. The boxed areas are magnified (lower right insets) to show apoptotic cells. The number of apoptotic cells between the dotted lines in each section was counted. **D and E**, Quantification of the number of apoptotic cells detected in panels A, B, and C. The percentage of apoptotic cells per all cells between the dotted lines in *Nestin;Epha4* ^*fx/fx*^ (D) and *GFAP;Epha4* ^*fx/fx*^ (E) mice is shown. There was no significant difference in the percentage of apoptotic cells. N = 4 per genotype. Error bars represent SD. Scale bar, 100 μm.

The number of apoptotic cells detected by TUNEL staining was similar to controls at E14.5 in both mutants (Fig 4). These findings suggest that neurogenesis was temporarily accelerated around E14.5 by *Epha4* deletion, regardless of the timing of the deletion (E11.5 or E13.5), but that RGC self-renewal after E17.5 was impaired by earlier deletion of *Epha4*, resulting in RGC depletion and eventual reduction of cortical thickness at P0.

**Fig 5:**
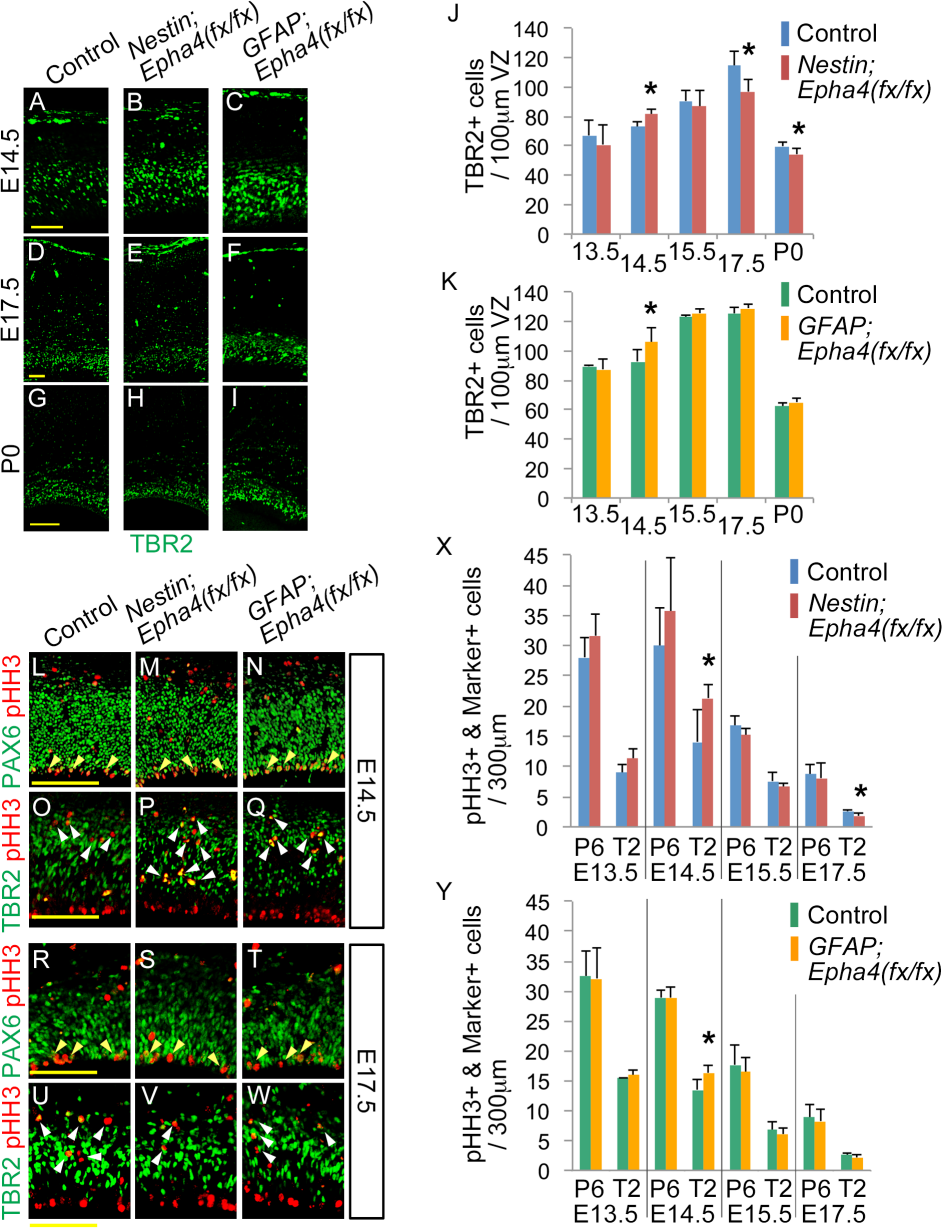
Production of TBR2+ INPs and pHH3+ mitotic cells in the VZ and SVZ of *Nestin;Epha4* ^*fx/fx*^ and *GFAP;Epha4* ^*fx/fx*^ mice. **A–I,** Anterior sections isolated from mutants and control mice between E13.5 and P0 were immunostained for an INP marker (TBR2, green). **J and K,** The number of TBR2+ cells within 100-μm-wide regions of *Nestin;Epha4* ^*fx/fx*^ (J) and *GFAP;Epha4* ^*fx/fx*^ (K) mouse cortex. At E14.5, TBR2 immunostaining revealed overproduction of INPs in both mutants compared to controls (A–C, J, K), suggesting that there may be accelerated cortical neurogenesis around E14.5 in both mutants. However, at E17.5 and P0, TBR2+ cells were depleted in *Nestin;Epha4* ^*fx/fx*^ mice (E, H, E17.5 and P0 in J) but the numbers were unaffected in *GFAP;Epha4* ^*fx/fx*^ mice compared to controls (F, I, E17.5 and P0 in K). **L–W,** Co-staining for pHH3 and PAX6 or TBR2. Mitotic cells were immunostained for pHH3 (red). The pHH3+ cells were co-stained with PAX6 (green), a marker for RGCs, or with TBR2 (green), a marker of INPs. Co-stained cells are shown by yellow and white arrowheads, respectively. **X and Y,** Quantification of the number of pHH3+ cells co-labeled by either marker in *Nestin;Epha4* ^*fx/fx*^ (X) and *GFAP;Epha4* ^*fx/fx*^ (Y) mice. The number of pHH3+PAX6+ RGCs (P6) and pHH3+TBR2+ INPs (T2) was counted separately at E13.5, E14.5, E15.5, and E17.5 in control, *Nestin;Epha4* ^*fx/fx*^, and *GFAP;Epha4* ^*fx/fx*^ mice. More INPs (pHH3+TBR2+) are dividing at E14.5 in both mutants. *Nestin;Epha4* ^*fx/fx*^ exhibited fewer mitotic RGCs (pHH3+PAX6+) than controls at E17.5, whereas *GFAP;Epha4* ^*fx/fx*^ mice showed no difference from controls. P6, pHH3+PAX6+; T2, pHH3+TBR2+. N = 5 (A–K) and 4 (L–Y), (*)P < 0.05. Error bars represent SD. Scale bars: A–F, 50 μm, G–I and L–W, 100 μm.

Many upper-layer cortical neurons are generated from TBR2+ INPs in the SVZ rather than directly from RGCs [6, 8, 35, 36]. To distinguish whether overproduction of cortical neurons at E14.5 is due to accelerated neuronal differentiation of RGC or enhanced transition to INPs, we examined the number of TBR2+ cells in the VZ and SVZ from E13.5 to P0. The number of TBR2+ cells increased transiently compared to control littermates at E14.5 in both mutants (Fig 5A–5C, 5J and 5K), suggesting enhanced proliferation of INPs at this stage. However, at later embryonic stages, the number of TBR2+ INPs decreased in *Nestin;Epha4* ^*fx/fx*^ mice but remained at control levels in *GFAP;Epha4* ^*fx/fx*^ mouse cortex (Fig 5D–5K). We next quantified the number of mitotic progenitor cells labeled with anti-phosphohistone-H3 (pHH3) antibody from E13.5 to E17.5 based on their co-staining with PAX6 (RGCs) or TBR2 (INPs). In both mutants, the number of mitotic cells at E14.5 increased in TBR2+ INPs, but not in PAX6+ RGCs, suggesting that replication of INPs is accelerated at this stage regardless of the time of *Epha4* deletion (Fig 5L–5Q, 5X and 5Y). The number of pHH3+TBR2+ cells at E17.5 was significantly below the control level in *Nestin;Epha4* ^*fx/fx*^ mice but not significantly different from control levels in *GFAP;Epha4* ^*fx/fx*^ mice (Fig 5R–5Y).

The results of these INP cell-marking studies using TBR2 or pHH3 were consistent at E14.5, indicating that deletion of EphA4 leads to overproduction of neurons due to a greater precursor population (of INPs). One tentative interpretation is that EphA4 normally suppresses the asymmetrical division of RGCs toward the INP lineage and the replication of INPs, in addition to augmenting RGC self-renewal. Later in development (E17.5–P0), the number of INPs and RGCs is reduced in *Nestin;Epha4* ^*fx/fx*^ mice compared to controls, but not in *GFAP;Epha4* ^*fx/fx*^ mice. This difference may be explained by the stage-dependent effect of EphA4 deletion on RGC and INP proliferation and differentiation.

### EphA4 is required for maintaining the self-renewal of RGCs and suppressing differentiation to the neuronal lineage

**Fig 6:**
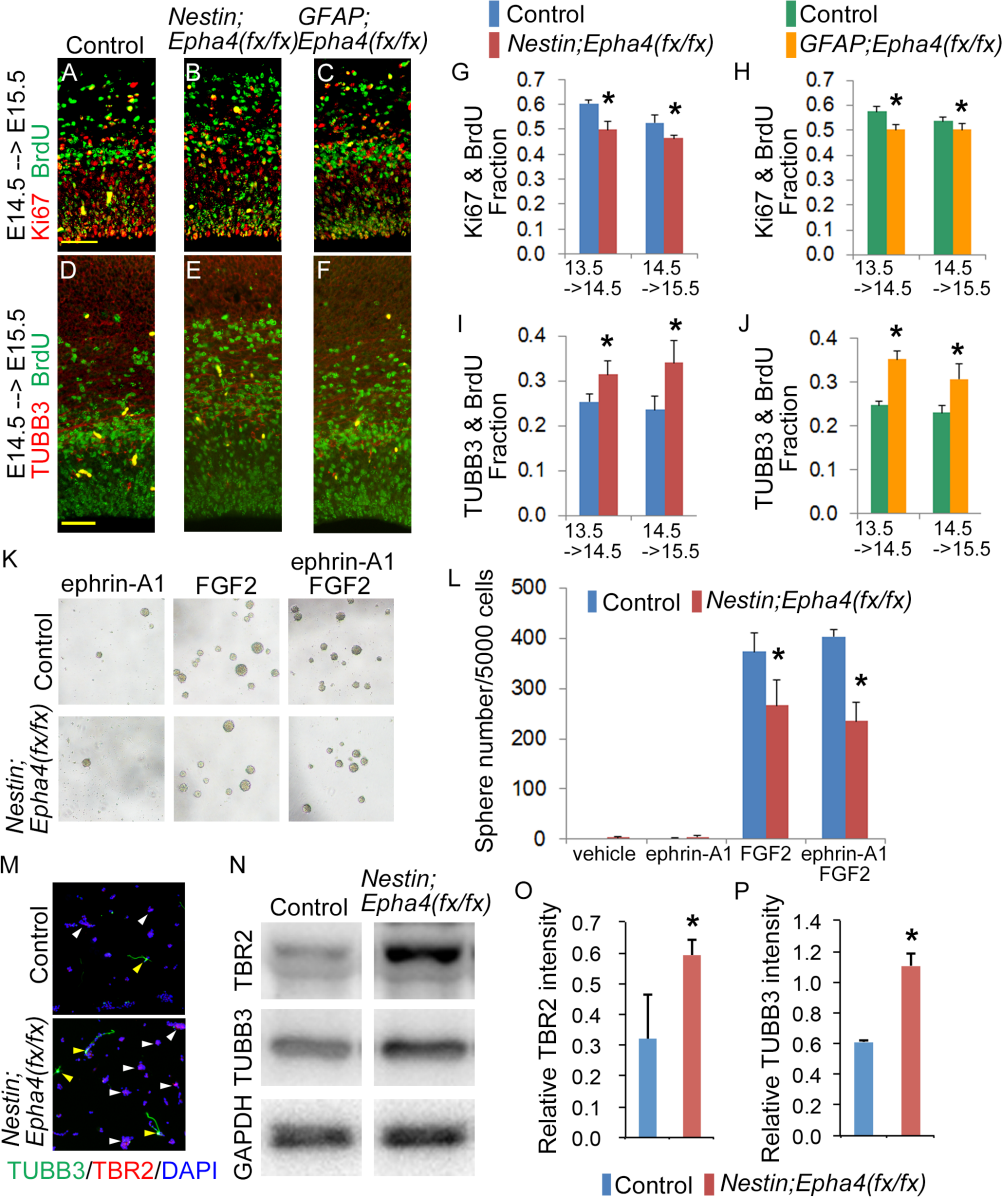
Self-renewal and differentiation of neural stem/progenitor cells. **A–J,** Evaluation of the population of cells that re-enter and exit the cell cycle in *Nestin;Epha4* ^*fx/fx*^ and *GFAP;Epha4* ^*fx/fx*^ mice between E13.5 and E15.5. BrdU was administered to pregnant mothers 24 h before collecting E14.5 and E15.5 embryos. Coronal sections of the control and mutant cortices were co-stained for BrdU and Ki67 to detect the cell population that re-enters the cell cycle and co-stained for BrdU and TUBB3 to mark the cell population that exits the cell cycle. Ki67+BrdU+ cells represent proliferating cells that include RGCs and INPs, while TUBB3+BrdU+ cells represent differentiated neuronal cells. Representative immunofluorescent images at E15.5 for BrdU (green) and Ki67 (red) (A–C), and those for BrdU (green) and TUBB3 (red) (D–F). Quantification of the number of Ki67+BrdU+ cells (G, H) and TUBB3+BrdU+ cells (I, J) in *Nestin;Epha4* ^*fx/fx*^ (G, I) and *GFAP;Epha4* ^*fx/fx*^ (H, J) mice. In both mutants, the Ki67+BrdU+ cell to total BrdU+ cell ratio decreased around E14.5, whereas the TUBB3+BrdU+ cell to total BrdU+ cell ratio significantly increased relative to controls. These findings suggest that differentiation of RGCs and INPs to neurons is accelerated at this stage in mutants relative to controls. These changes are expressed quantitatively as the fraction of double stained cells relative to the total number of BrdU+ cells in the cortex. N = 5 per genotype, (*) P < 0.05. Error bars represent SD. Scale bars, 50 μm. **K and L,** Neurosphere formation from cortical cells isolated from *Nestin;Epha4* ^*fx/fx*^ cortex in the presence of FGF2 alone, clustered ephrin-A1 alone, or FGF2 plus clustered ephrin-A1. Representative images of neurosphere formation (K) and the number of neurospheres in K (L). Fewer neurospheres grew from isolated *Nestin;Epha4* ^*fx/fx*^ cortical cells than from control cortical cells. FGF2 increased the number of the neurospheres derived from control E14.5 cortex but not that from mutant E14.5 cortex, whereas clustered ephrin-A1 alone, a ligand for EphA4, did not affect neurosphere growth. **M–P,** Differentiation of neurospheres derived from control and *Nestin;Epha4* ^*fx/fx*^ cortical cells. The cells from the E14.5 cortex were cultured for 4–5 days to form primary neurospheres. Cells from the primary neurospheres were replated to obtain a monolayer culture and used for immunostaining as described in the Materials and Methods. Representative image of neurospheres immunostained with markers for INPs (TBR2, red) and neurons (TUBB3, green) (M). There was a higher percentage of spheres with TBR2+ (white arrowheads) and TUBB3+ (yellow arrowheads) cells in the mutant (lower figure in M) than in controls (upper figure in M). Representative western blot analysis of TBR2 and TUBB3 in neurospheres from control and *Nestin;Epha4* ^*fx/fx*^ mice (N) and quantification of TBR2 (O) and TUBB3 (P) signal intensities in neurospheres from control and *Nestin;Epha4* ^*fx/fx*^ mice. The level of TBR2 and TUBB3 expression in the spheres increased in mutants compared to controls, showing that spheres of *Nestin;Epha4* ^*fx/fx*^ are in a more differentiated state compared to control. N = 5 per genotype, (*) P < 0.05. Error bars represent SD.

Despite accelerated generation of TUBB3+ neurons in both mutants, the number of PAX6+ cells and the radial thickness of the PAX6+ cell layer were unchanged until around E14.5 (Fig 3J–3Q). In *Nestin;Epha4* ^*fx/fx*^ mice, however, these parameters, reflecting RGC proliferation and differentiation, were reduced after E17.5 compared to controls (Fig 3D, 3E, 3G, 3H, 3J and 3L). The number of INPs also decreased after E17.5 (Fig 5J and 5X), and the number and thickness of the cortical layer of post-mitotic neurons were considerably reduced by P0 (Figs 3G, 3H, 3N, 3P and 5J). Since the decrease in the number of INPs and cortical neurons could result from a reduced number of RGCs (the common precursor), we speculated that early accelerated differentiation to cortical neurons leads to depletion of RGCs in these mice. To find more direct evidence for decreased self-renewal and increased differentiation of RGCs in the VZ, we quantified the number of cells committed to cell division and the number of post-mitotic neurons. Mitotically active precursor cells were labeled with a pulse of BrdU at E13.5 or E14.5, and the fate of these mitotic cell populations was assessed 24 h later by cell-selective immunostaining. Incorporation of BrdU into dividing precursor cells was examined in combination with Ki67, a protein marker of dividing cells, or with TUBB3, a marker of post-mitotic neurons, to determine cell cycle reentry or exit, respectively, by cells that were mitotic at the time of the BrdU pulse. In mice labeled at either E13.5 or E14.5, we found a significant decrease in the Ki67+BrdU+ cell to total BrdU+ cell ratio in both mutants compared to controls, indicating that RGC self-renewal is diminished by both early and later *Epha4* deletion (Fig 6A–6C, 6G and 6H). Consistent with these findings, the TUBB3+BrdU+ cell to total BrdU+ cell ratio increased, indicating elevated production of post-mitotic neurons in both EphA4 mutants concomitant with reduced RGC self-renewal (Fig 6D–6F, 6I and 6J).

To confirm this shift from RGC self-renewal to neuronal differentiation in mutant mice, we examined the capacity of isolated cortical cells to produce neurospheres *in vitro*. Embryonic day 14.5 cortical cells from control mice and mutants were dissociated and cultured in the presence of FGF2 alone, clustered ephrin-A1 alone, or FGF2 plus clustered ephrin-A1 [37]. As described above, EphA4 expression was detected in both the basal ganglia and the cortex of *GFAP;Epha4* ^*fx/fx*^ mice. To avoid contamination by EphA4+ cells, we used cells only from *Nestin;Epha4* ^*fx/fx*^ cortex for this experiment. Control E14.5 cortical cells developed into a larger number of neurospheres than E14.5 cortical cells from *Nestin;Epha4* ^*fx/fx*^ mice in the presence of either FGF2 alone or FGF2 plus clustered ephrin-A1 (Fig 6K and 6L). In addition to neurosphere formation, we examined the effect of EphA4 deletion on the neuronal differentiation of neurospheres using immunostaining. Neurospheres derived from the mutant formed more TBR2+ cells and TUBB3+ cells than control neurospheres (Fig 6M). We also confirmed the increased expression level of TBR2 and TUBB3 in mutant spheres by western blotting analysis (Fig 6N–6P). These results suggest that EphA4 is required for FGF2-mediated self-renewal of neural stem cells/progenitors in culture.

These results indicate that EphA4 appears critical for RGC self-renewal around E14.5. *Epha4* deletion induces premature progression of RGCs toward the neuronal cell lineage, and this accelerated differentiation to cortical neurons during early corticogenesis results in depletion of RGC and, ultimately, in a reduced cortical volume by P0.

### Early loss of *Epha4* leads to aberrant corticogenesis via precocious neuronal differentiation

**Fig 7:**
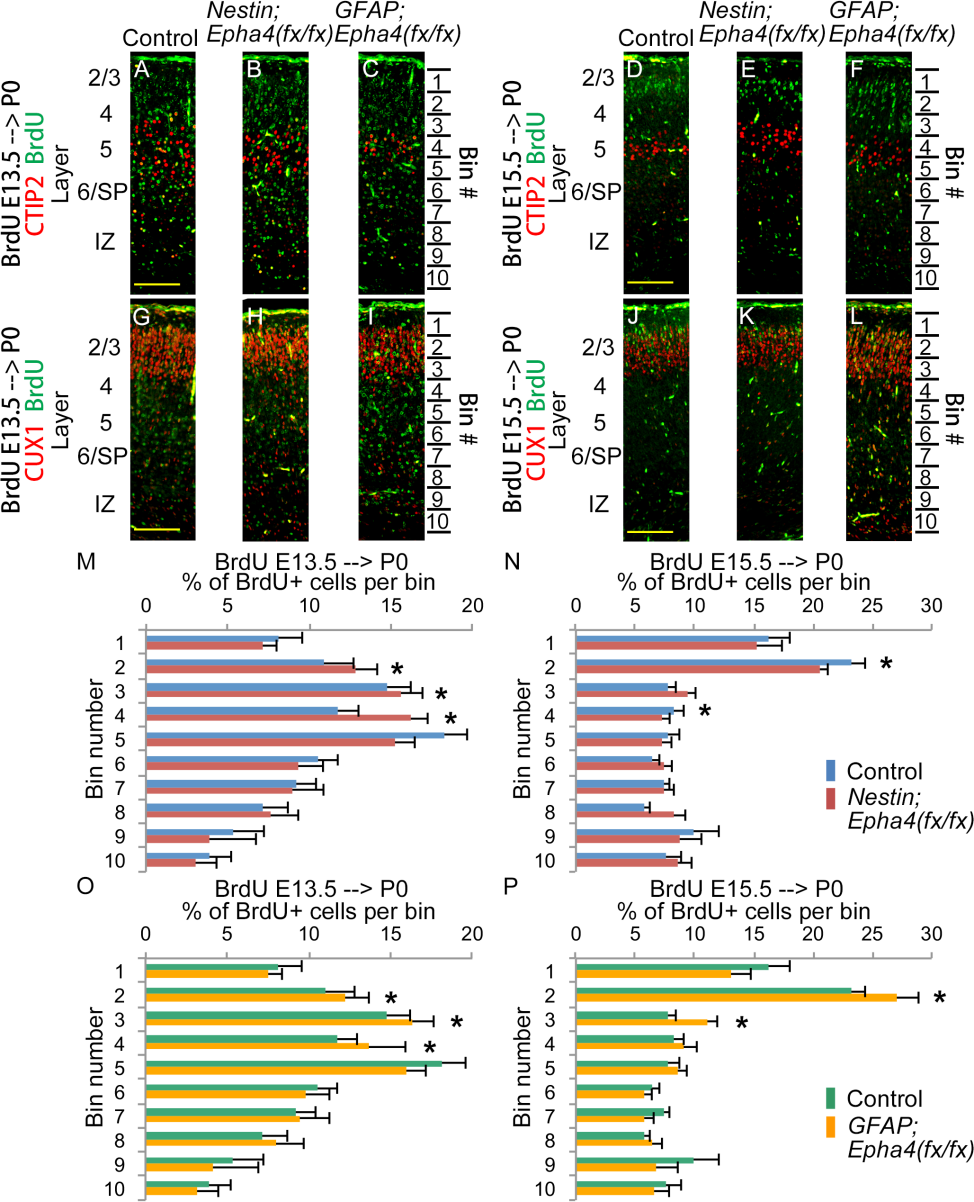
Positioning of cortical neurons born at E13.5 and E15.5 in P0 mice. **A–L,** Representative immunofluorescent images showing the cortical distribution of BrdU-labeled cells (green) and markers (red) for layer 5 (CTIP2) and layers 2/3 (CUX1). Layer names are shown on the left of the images. **M–P,** Quantified distribution of BrdU+ cell numbers in 10 bins spanning the layers of the cortex. BrdU was injected to label proliferative cells that differentiate into migrating neurons at E13.5 (A–C, G–I, M, O) and E15.5 (D–F, J–L, N, P). The cortical distributions of these cells were analyzed at P0 in control (A, D, G, J), *Nestin;Epha4* ^*fx/fx*^ (B, E, H, K) and *GFAP;Epha4* ^*fx/fx*^ (C, F, I, L) mice. In *Nestin;Epha4* ^*fx/fx*^ mice, a greater fraction of neurons born at E13.5 (M, red bar) was found in upper cortical layers compared to controls (M, blue bar), but a smaller fraction of cells born at E15.5 (N, red bar) was found in upper layers compared to controls (N, blue bar). In contrast, *GFAP;Epha4* ^*fx/fx*^ mice exhibited a greater fraction of neurons born at both E13.5 and E15.5 (O and P, yellow bar) in upper cortical layers compared to controls (O and P, green bar). N = 3 per genotype, (*) P < 0.01. Error bars represent SD.

The early transition of RGCs to INPs and neurons in the VZ of *Epha4* deletion mutants around E14.5 could conceivably alter the laminar structure of the cerebral cortex. The laminar pattern of cortical neurons is generated by two developmental processes, migration of neurons to their final destination within the nascent neocortex and acquisition of distinct neuronal identities [5, 38]. We first examined whether RGCs lacking EphA4 generate cortical layers through neural migration in *Nestin;Epha4* ^*fx/fx*^ and *GFAP;Epha4* ^*fx/fx*^ mutants. Since the neurons born at E13.5 are predominantly deep layer neurons and those born at E15.5 are upper-layer neurons [39], we performed birthdating experiments by injecting mice with BrdU at E13.5 or E15.5 [40]. EphA4 was deleted by E13.5 in both mutants, as described above (Fig 2). We divided the P0 cortex into 10 subregions of equivalent thickness (bins) and measured the fraction of BrdU+ cells in each. *Nestin;Epha4* ^*fx/fx*^ mice labeled with BrdU at E13.5 showed a significant increase in the number of BrdU+ neurons in bins 2–4 compared to controls (Fig 7A, 7B, 7G, 7H and 7M). However, the same mutant mice labeled at E15.5 showed a significant decrease in the number of BrdU+ cells in bins 2 and 4 (Fig 7D, 7E, 7J, 7K and 7N). *GFAP;Epha4* ^*fx/fx*^ mice labeled at E13.5 showed an increase in the number of BrdU+ neurons in bins 2–4, similar to the findings in *Nestin;Epha4* ^*fx/fx*^ mice, but also showed an increase in the number of BrdU+ cells in bins 2 and 3 when labeled at E15.5 (Fig 7A, 7C, 7G, 7I, 7D, 7F, 7J, 7L, 7O and 7P). These findings suggest that the migration bias toward the upper layer at E13.5 may be augmented in both mutants by generation of more upper-layer neurons at this time point. The difference in the number of E15.5-labeled BrdU+ neurons between *Nestin;Epha4* ^*fx/fx*^ and *GFAP;Epha4* ^*fx/fx*^ may be caused by the difference in *Epha4* deletion timing.

**Fig 8:**
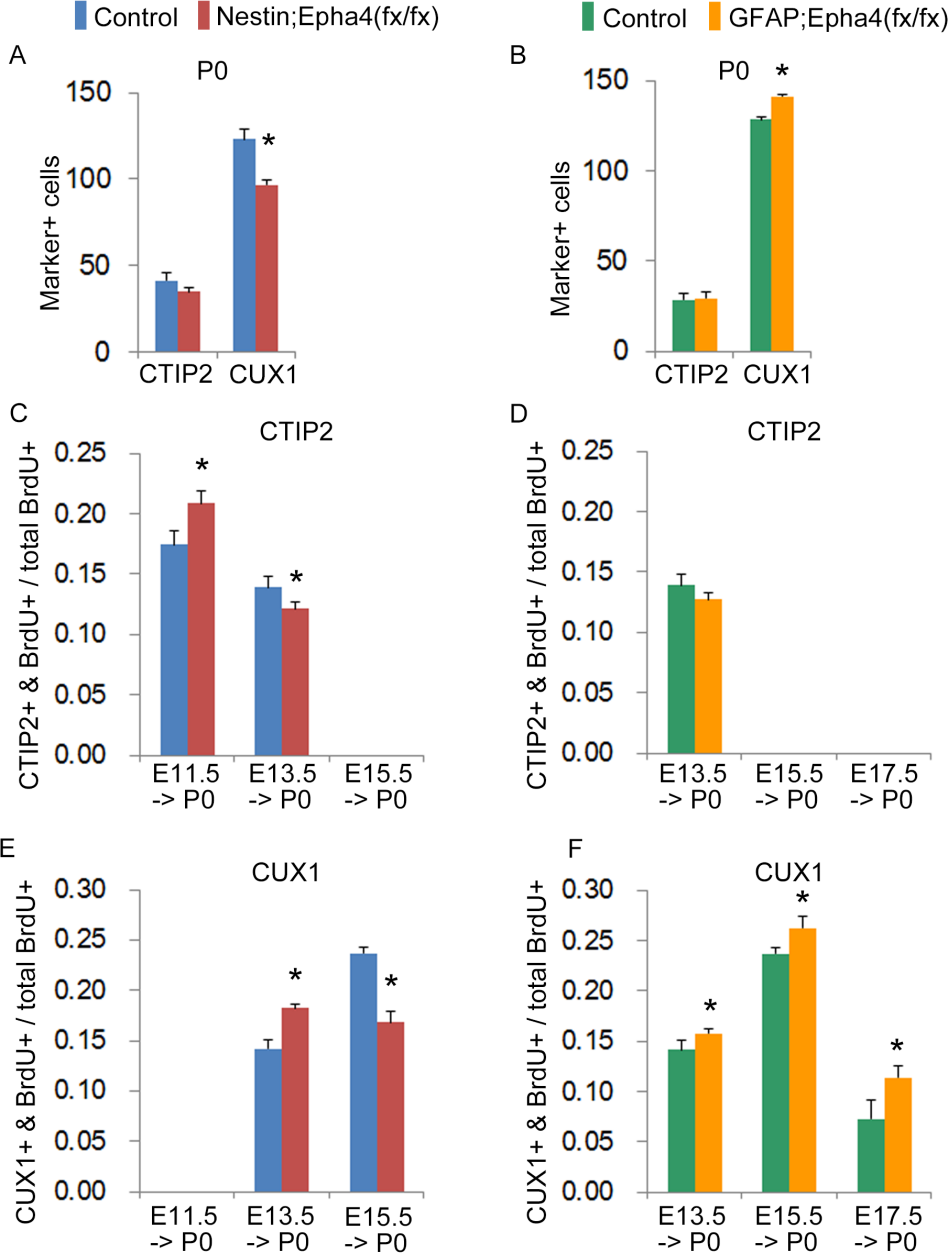
Cortical distribution of neurons born at E11.5, E13.5, E15.5, or E17.5 in P0 mice. **A and B,** Total number of cells labeled with the layer marker CTIP2 or CUX1 at P0 in *Nestin;Epha4* ^*fx/fx*^ (A) or *GFAP;Epha4* ^*fx/fx*^ (B) mice compared to controls. **C**–**F**, The ratio of the number of CTIP2+BrdU+ or CUX1+BrdU+ cells relative to the total number of BrdU+ cells. BrdU was injected to label proliferative cells at E11.5, E13.5, E15.5, and E17.5 in *Nestin;Epha4* ^*fx/fx*^ (C, E), *GFAP;Epha4* ^*fx/fx*^ (D, F) and corresponding control mice. Both the mutant and the control embryos show a similar cortical layer structure. However, the total number of CUX1+ cells at P0 decreased in *Nestin;Epha4* ^*fx/fx*^ (A) but increased in *GFAP;Epha4* ^*fx/fx*^ (B) mice compared to controls. In *Nestin;Epha4* ^*fx/fx*^ mice, a larger number of CTIP2+ cells was co-labeled with BrdU at E11.5 but fewer were co-labeled at E13.5 compared to controls (C), whereas in *GFAP;Epha4* ^*fx/fx*^ mice, the number of co-labeled cells was similar to that of controls at both E11.5 and E13.5 (D). The number of CUX1+ cells co-labeled with BrdU at E13.5 was greater in both mutants (E, F), while the number at E15.5 was lower in *Nestin;Epha4* ^*fx/fx*^ mice (E) and greater in *GFAP;Epha4* ^*fx/fx*^ mice compared to controls (F). IZ, intermediate zone; SP, subplate. N = 6 for total number of CUX1+ cells and N = 3 for other markers per genotype, (*) P < 0.01. Error bars represent SD.

We then examined the relative proportion of cells that acquired a specific layer fate by immunostaining with specific markers for layers 2/3 (CUX1) or layer 5 (CTIP2) in the dorsolateral neocortex [39]. CUX1+ and CTIP2+ cells in mutant mice were, similar to cells in control mice, distributed in layers 2/3 and 5, respectively (Fig 7A–7L), suggesting that there is no apparent disturbance in the fate of cortical neuronal cells in the absence of EphA4. However, we observed a significant decrease in the number of CUX1+ cells in *Nestin;Epha4* ^*fx/fx*^ mice compared to controls at P0 (Fig 8A), while *GFAP;Epha4* ^*fx/fx*^ mice showed a significant increase in the number of CUX1+ cells at P0 (Fig 8B).

We therefore examined the identity of neurons born at each stage in *Epha4* mutants. We quantified the ratio of neurons born at a given developmental stage relative to the total number of BrdU+ cells within both upper and lower cortical layers of mutants and controls. The dividing cells labeled by BrdU at E11.5, E13.5, E15.5, or E17.5 were examined at P0 for BrdU and a layer-specific marker, CTIP2 for layer 5 or CUX1 for layers 2/3, in anterior brain sections. *Nestin;Epha4* ^*fx/fx*^ mice showed a significant increase in the ratio of CTIP2+BrdU+ neurons to total BrdU+ cells born at E11.5, a significant increase in the ratio of CUX1+BrdU+ neurons to total BrdU+ cells born at E13.5, and a significant decrease in the ratio of CTIP2+BrdU+ neurons to total BrdU+ cells born at E13.5 (Fig 8C and 8E). The same mutant mice labeled with BrdU at E15.5 also showed a significant decrease in the number of CUX1+BrdU+ neurons and no detectable CTIP2+BrdU+ cells (Fig 8C and 8E). Therefore, deep layer neurons expressing CTIP2 appear to be generated earlier in *Nestin;Epha4* ^*fx/fx*^ mice than in controls, again suggesting precocious neurogenesis. Since neurogenesis in *Nestin;Epha4* ^*fx/fx*^ mice is accelerated after E11.5, the stage of EphA4 deletion in this mutant, precocious differentiation to neurons may account for depletion of PAX6+ RGCs at P0 in *Nestin;Epha4* ^*fx/fx*^ mice (Figs 1N, 3J, 3L, 8C and 8E). In the case of *GFAP;Epha4* ^*fx/fx*^ mice, however, the ratio of CTIP2+BrdU+ neurons to total BrdU+ cells born at E13.5, the stage of EphA4 deletion in these mutants, remained unchanged (Fig 8D), suggesting that the deep layer neurons expressing CTIP2 had already completed migration by this stage. Furthermore, in contrast to the results in *Nestin;Epha4* ^*fx/fx*^ mice, *GFAP;Epha4* ^*fx/fx*^ mice showed a significant increase in the ratio of CUX1+BrdU+ neurons born at all of the stages, including E13.5, E15.5, and E17.5 (Fig 8F).

### EphA4 regulates cortical neurogenesis by modulating an FGF signal

**Fig 9:**
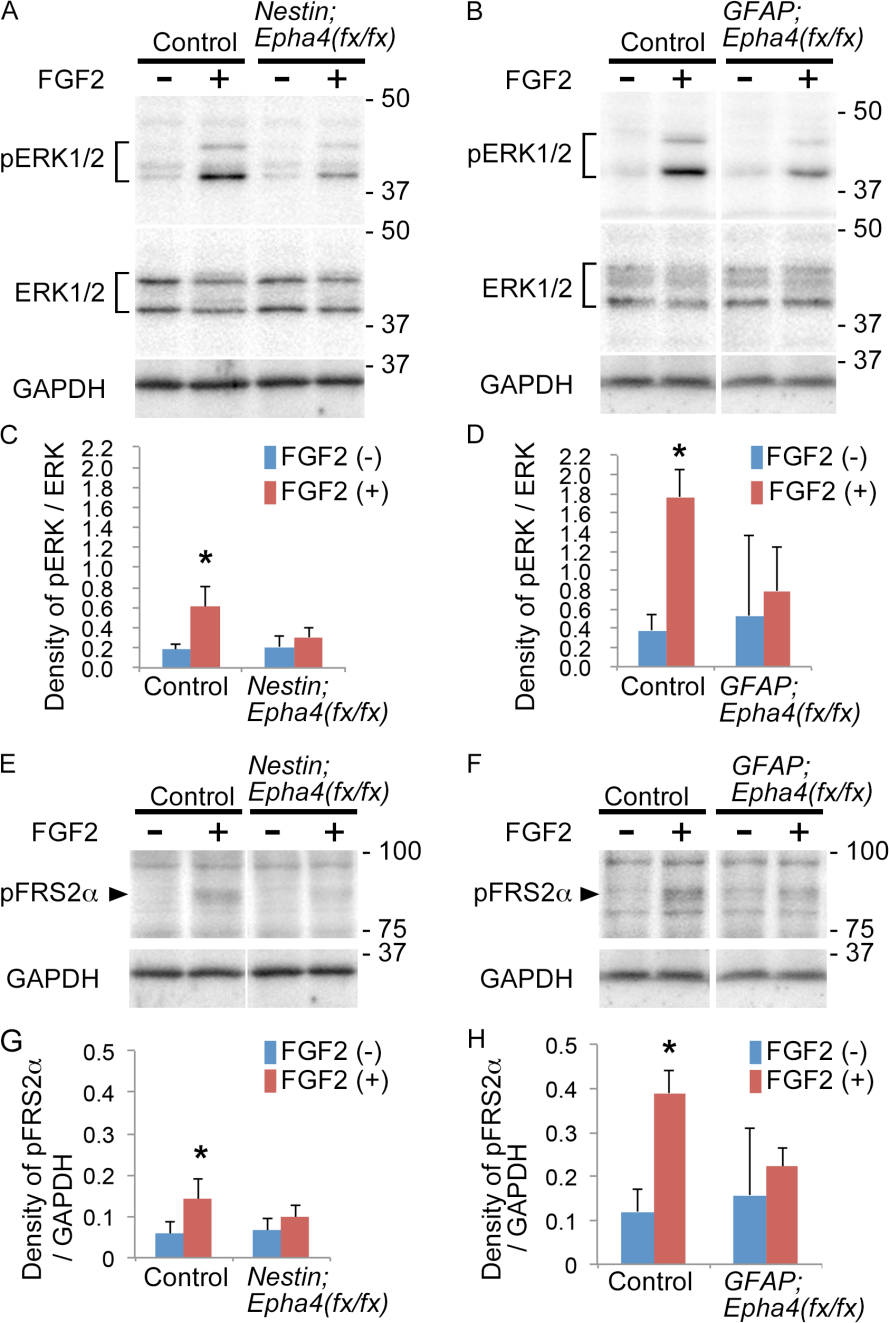
ERK1/2 and FRS2α phosphorylation in cortical cells in response to FGF2. **A and B,** Representative western blot analysis of ERK1/2 phosphorylation (pERK1/2) in cortical cells from control and *Nestin;Epha4* ^*fx/fx*^ mice (A) or *GFAP;Epha4* ^*fx/fx*^ mice (B). Dissociated cells from the E15.5 telencephalon were stimulated for 20 min with FGF2 after 1 h starvation and lysed immediately. Stimulated and non-stimulated cells are marked by + or—, respectively. Total ERK1/2 expression (ERK) was used to show equal sample loading. **C and D,** Quantification of pERK1/2 and ERK1/2 signal intensities in the cortical cells stimulated with FGF2 (+) or not (-) in *Nestin;Epha4* ^*fx/fx*^ (C) and *GFAP;Epha4* ^*fx/fx*^ (D) mice. **E and F,** Representative western blot analysis of FRS2α phosphorylation (pFRS2α) in cortical cells from control and *Nestin;Epha4* ^*fx/fx*^ mice (E) or *GFAP;Epha4* ^*fx/fx*^ mice (F). Cells from the E15.5 telencephalon were examined as described above. GAPDH expression was used to show equal sample loading. **G and H,** Quantification of pFRS2α and GAPDH signal intensities in cortical cells stimulated with FGF2 (+) or not (-) in *Nestin;Epha4* ^*fx/fx*^ (G) and *GFAP;Epha4* ^*fx/fx*^ (H) mice. ERK1/2 and FRS2α phosphorylation levels were much lower in both mutants compared to controls. Molecular size markers are shown in kDa on the right side of the western blot lanes. N = 5 per genotype, (*) P < 0.05. Error bars represent SD.

We reported previously that EphA4 forms a ternary complex with FGFRs and FRS2α and augments the canonical FGF signal through tyrosine phosphorylation of FRS2α and ERK activation [18, 41]. To examine whether loss of EphA4 affects the FGF signaling pathway in the cortex, western blotting was used to examine the activation of downstream FGF signaling molecules, phosphorylated FRS2α (pFRS2α) and ERK1/2 (pERK1/2), in response to FGF2 in E15.5 *Epha4* mutants and control mice. Both FRS2α and ERK1/2 were phospho-activated in response to FGF2 stimulation in the control, but these proteins in the mutants were much less activated than in the control (Fig 9), suggesting that loss of EphA4 diminishes FGF signaling in cortical cells.

## Discussion

Most developmental processes, including proliferation, survival, apoptosis, migration, metabolic alterations, and morphological changes, are triggered by synthesis of signaling molecules and binding to distinct receptors expressed by neighboring cells. The Eph/ephrin cell-cell contact-mediated signaling pathway is implicated in many biological events [16, 42]. In brain development, EphA4 signaling is involved in the control of embryonic and postnatal neurogenesis [25, 26]. Multiple FGF signaling pathways participate in corticogenesis. At least 8 of the 22 members of the FGF ligand family, FGF2, 3, 7, 8, 10, 15, 17, and 18, are expressed in early forebrain and function in mammalian corticogenesis by binding to and activating three distinct FGFRs, FGFR1, FGFR2, and FGFR3 [14, 43–47]. A few molecules, such as heparan sulfate proteoglycans [48], Klotho [49], XFLRT3 [50], SEF [51], and EphA4 [18, 41], are known to modulate FGF signaling by binding to FGF ligands and/or FGFRs.

During cortical neurogenesis, RGCs not only self-renew but also generate cortical neurons by undergoing asymmetrical division to INPs and further differentiation through this lineage. Peak neurogenesis occurs around E14.5 [52]. We generated two lines of *Epha4* deletion mutant mice using Nestin-Cre and GFAP-Cre. These mice show loss of EphA4 expression in a stage-specific manner, after E11.5 by Nestin-Cre (*Nestin;Epha4* ^*fx/fx*^) and after E13.5 by GFAP-Cre (*GFAP;Epha4* ^*fx/fx*^). We found a similar phenotype in both mutants around E14.5 that suggested upregulated neurogenesis. The number of TBR2+ or pHH3+ mitotic cells in the SVZ, representing INPs, increased significantly at E14.5 compared to controls, but the number of PAX6+ RGCs remained constant, suggesting that EphA4 acts to repress both replication of INPs and differentiation of INPs to neurons at this stage without an apparent effect on RGC cell division.

However, *Nestin;Epha4* ^*fx/fx*^ and *GFAP;Epha4* ^*fx/fx*^ mice exhibited distinct phenotypes at P0. Overproduction of neurons at E14.5 was not maintained after E15.5 in either mutant. In *Nestin;Epha4* ^*fx/fx*^ mice, the number of RGCs expressing PAX6 in the VZ and INPs expressing TBR2 in the SVZ decreased at E17.5 and P0. The number of mitotic cells labeled by pHH3 in the SVZ of *Nestin;Epha4* ^*fx/fx*^ mice was also reduced at E17.5 compared to control mice. Moreover, in P0 cortex, *Nestin;Epha4* ^*fx/fx*^ mice produced more upper-layer CUX1+ neurons born at E13.5 than controls, while the number of CUX1+ neurons born at E15.5 was reduced. These results suggest precocious neurogenesis and subsequent depletion of RGCs in *Nestin;Epha4* ^*fx/fx*^ mice. This conclusion was supported by neurosphere formation experiments and analysis of neurospheres for differentiation. Cells isolated from the E14.5 cortex of *Nestin;Epha4* ^*fx/fx*^ mice formed fewer neurospheres in response to FGF2 than controls, and increased expression of the markers for INPs (TBR2) and neurons (TUBB3) compared to controls, indicating that the commitment and progression from RGCs to INPs and/or from INPs to neurons have already begun at this stage. Hence, EphA4 functions to maintain RGC self-renewal and repress the differentiation of RGCs to INPs and INPs to cortical neurons.

In contrast to *Nestin;Epha4* ^*fx/fx*^ mice, the number of RGCs, INPs, and mitotic cells in *GFAP;Epha4* ^*fx/fx*^ mice at E17.5 and P0 was similar to controls. In addition, the number of CUX1+ neurons born at E13.5, E15.5, and E17.5 increased in the cortex of this mutant at P0, suggesting that the precocious neurogenesis and depletion of RGCs observed in *Nestin;Epha4* ^*fx/fx*^ mice does not occur in *GFAP;Epha4* ^*fx/fx*^ mice. However, there were more RGCs exiting the cell cycle and fewer re-entering the cell cycle at E14.5 in *GFAP;Epha4* ^*fx/fx*^ mice than controls, suggesting that *GFAP;Epha4* ^*fx/fx*^ mice also show temporarily accelerated neurogenesis, similar to *Nestin;Epha4* ^*fx/fx*^ mice, at this developmental stage.

The distinct cortical phenotypes of the two mutants at P0 may be caused by the difference in the developmental stage when EphA4 is deleted. RGCs extensively expand their cell pool before E11.5 and begin to deplete the pool after E11.5, when neurogenesis occurs. By E11.5, *Epha4* had been deleted in *Nestin;Epha4* ^*fx/fx*^ mice but not in *GFAP;Epha4* ^*fx/fx*^ mice. *Epha4* deletion in *GFAP;Epha4* ^*fx/fx*^ mice occurs 2 days later (E13.5) than in *Nestin;Epha4* ^*fx/fx*^ mice. Thus, at the time when neurogenesis starts, *GFAP;Epha4* ^*fx/fx*^ mice have a more abundant RGC pool than *Nestin;Epha4* ^*fx/fx*^ mice. The delay in *Epha4* deletion in *GFAP;Epha4* ^*fx/fx*^ mice may be why the decrease in RGCs is not observable by P0. The decrease in the number of RGCs in *Nestin;Epha4* ^*fx/fx*^ mice was detectable at E17.5, even though precocious neurogenesis had already been observed in *Nestin;Epha4* ^*fx/fx*^ mice at E11.5. Therefore, in *GFAP;Epha4* ^*fx/fx*^ mice, depletion of RGCs due to precocious neurogenesis might become detectable at a later stage than in *Nestin;Epha4* ^*fx/fx*^ mice.

By deleting EphA4 expression at different developmental stages in the cortex, we were able to identify three mechanisms determining cortex size at P0: (1) EphA4 functions to repress differentiation through the neuronal lineage, (2) EphA4 regulates the production of INPs from RGCs, and (3) EphA4 appears critical for maintaining the self-renewal of RGCs, especially before E13.5. Regarding this third point, however, the number of PAX6+ RGCs did not decrease until E17.5, even in *Nestin;Epha4* ^*fx/fx*^ mice. A recent study identified a new type of progenitor cells in mice, radial glial-like cells (oRGCs), which are likely a progeny of RGCs. oRGCs are PAX6+ but monopolar cells with only a basal process and without an apical process, in contrast to bipolar RGCs [53]. We, therefore, cannot rule out the possibility that the PAX6+ cell population in *Nestin;Epha4* ^*fx/fx*^ mouse cortex may have included a substantial number of oRGCs, and so obscured the accelerated differentiation of RGCs before E17.5.

We previously reported that trans-activation of EphA4 and FGFRs by a direct interaction phosphorylates a downstream docking protein, FRS2α, and activates ERK1/2 *in vitro* [18, 41]. We show in the current report that FRS2α and ERK1/2 are less activated by FGF2 in E15.5 cortical cells from both *Epha4* mutants compared to controls. Moreover, cortical cells isolated from mutant cortex formed fewer neurospheres than cells from control cortex in response to FGF2 stimulation. These results indicate that FGF responsiveness is reduced in cortical cells missing *Epha4*. Loss of FGF signaling by combined deletion of FGFR1, FGFR2, and FGFR3 in the cortex by E10 or E13.5 reduced PAX6+ RGCs and increased TBR1+ or TBR2+ INPs and TUBB3+ neurons [14, 15]. As these cortical phenotypes are similar to those of our *Epha4* mutants, we speculate that EphA4 supports RGC self-renewal and represses RGC differentiation into neurons, processes known to be mediated by FGF signaling [15, 54]. However, *Epha4* mutants did not show a measurable reduction in the number of RGCs or in the cortical area occupied by RGCs between E13.5 and E14.5, in contrast to FGFR mutants, which exhibited reductions in both RGC number and cortical area dominated by RGCs at this embryonic stage. Instead, we found a transient increase in INPs and differentiated neuronal cells at E14.5 in both *Epha4* mutants. Furthermore, cortical size at P0 was reduced in *Nestin;Epha4* ^*fx/fx*^ mice compared to *GFAP;Epha4* ^*fx/fx*^ mice. Taken together, these observations suggest that the balance between RGC self-renewal and the progression to more differentiated states is determined mainly by FGF signaling and regulated by EphA4 through complex formation with FGFRs *in vivo*. The function of EphA4 before E13.5 appears critical for determining the size of the cortex at P0.

**Fig 10:**
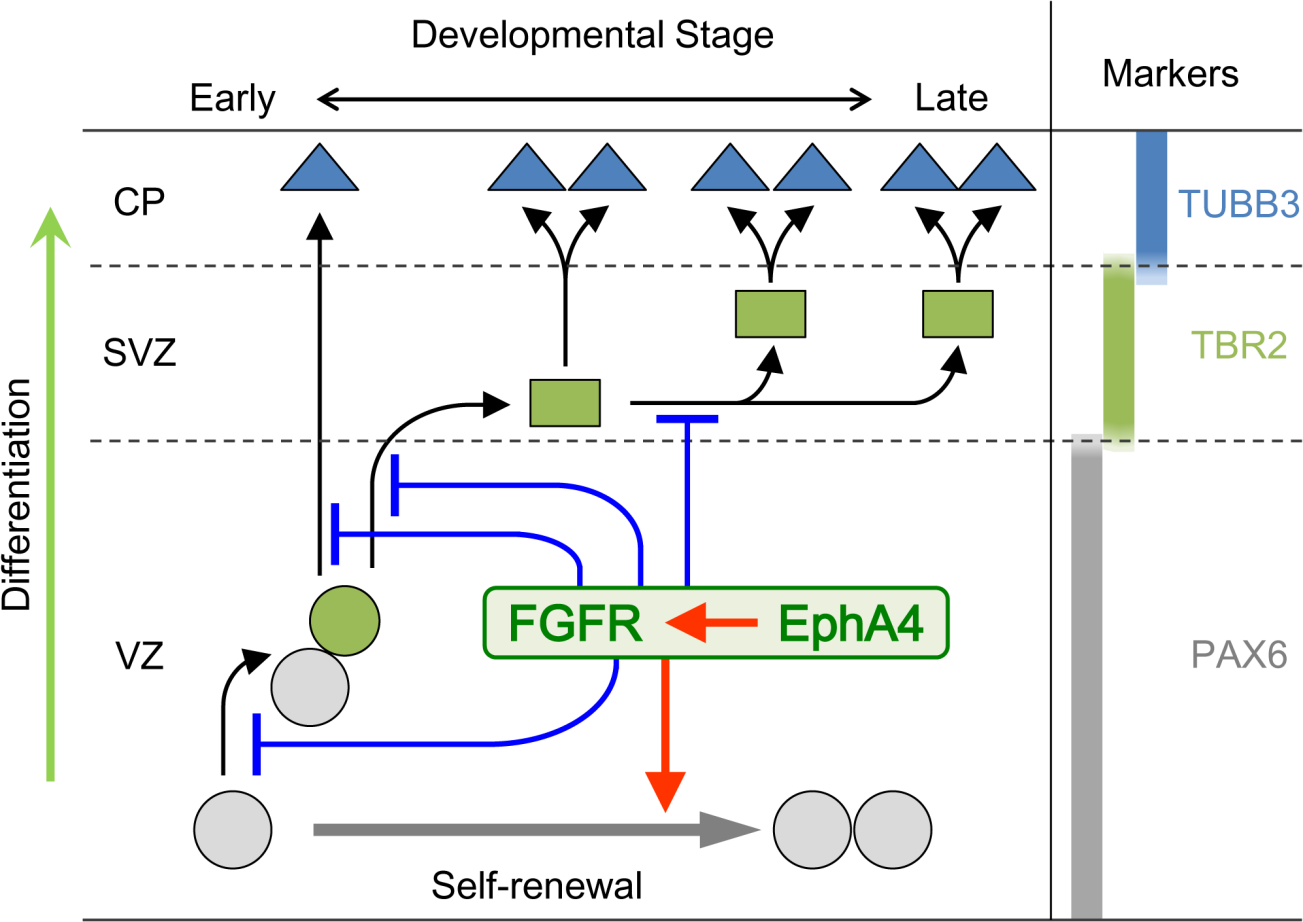
A model summarizing our results. In early brain corticogenesis, EphA4 in collaboration with FGFRs regulates transition through the neuronal lineage and accelerates self-renewal of RGCs. Black and gray arrows indicate the process of differentiation and self-renewal, respectively. Red arrow-headed lines indicate the function of acceleration and blue T-shaped lines indicate that of suppression. PAX6, TBR2, and TUBB3 are the markers for RGCs, INPs, and neuronal cells, respectively. Gray circles, RGCs; Green circle, committed RGCs; Green squares, INPs; Blue triangles, neuronal cells. CP, cortical plate; SVZ, subventricular zone; VZ, ventricular zone.

In summary, our study demonstrates that EphA4 plays an important role in modulating RGC self-renewal, INP proliferation, and the differentiation of RGCs and INPs through the neuronal lineage during embryonic corticogenesis (Fig 10). The regulatory function of EphA4 appears to be mediated by FGF signaling. The critical period for EphA4 expression that affects the size of the cortex at P0 occurs before E13.5, although disturbed expression later in development may also transiently influence cortical neurogenesis. However, gross cortical layer formation is not disturbed, even in the absence of EphA4 expression after E10.5.
